# Vestibular Adaptations Induced by Gentle Physical Activity Are Reduced Among Older Women

**DOI:** 10.3389/fnagi.2017.00167

**Published:** 2017-05-29

**Authors:** Julien Maitre, Thierry Paillard

**Affiliations:** Laboratoire Mouvement, Equilibre, Performance et Santé, Département STAPS, Université de Pau et des Pays de l’AdourTarbes, France

**Keywords:** postural control, exercise, sensory manipulation, galvanic vestibular stimulation, physical activity

## Abstract

The aim of this study was to compare the ability of older individuals to maintain an efficient upright stance in contexts of vestibular sensory manipulation, according to their physical activity status. Two groups of healthy older women (aged over 65) free from any disorders (i.e., neurological, motor and metabolic disorders) and vestibular disturbances, participated in this study. One group comprised participants who regularly practiced gentle physical activities, i.e., soft gym, aquarobic, active walking, ballroom dancing (active group, age: 73.4 (5.8) years, *n* = 17), and one group comprised participants who did not practice physical activities (non-active group, age: 73.7 (8.1) years, *n* = 17). The postural control of the two groups was compared in a bipedal reference condition with their eyes open and two vestibular sensory manipulation conditions (i.e., bipolar binaural galvanic vestibular stimulation (GVS) at 3 mA, in accordance with two designs). The main results indicate that there was no difference between the active and the non-active groups in all the conditions. It is likely that the aging process and the type of physical practice had limited the ability of the active group to counteract the effects of vestibular sensory manipulation on postural control more efficiently than the non-active group.

## Introduction

The human ability to maintain an efficient upright stance in daily life requires the availability and the accuracy of sensory, integrating and motor systems (Massion, 1994). As age increases, humans undergo an involution of these systems which leads to balance disorders (Sturnieks et al., 2008) and increases the risk of falls among older individuals (Lord and Sturnieks, 2005). Conversely, the beneficial effects of physical activity on postural function have been demonstrated for older individuals (Perrin et al., 1999; Howe et al., 2007) such as a better use of sensory information and a more efficient motor output (Gauchard et al., 2001; Ribeiro and Oliveira, 2007). Indeed, to some extent, physical activity performed on a regular basis may be able to protect the postural system from aging effects. To understand how aging and physical activity affect postural control, external perturbation techniques, more precisely sensory manipulation techniques (i.e., mechanical, electrical, chemical, optical) have been widely used (Gauchard et al., 2001, 2003; Hue et al., 2004; Jeka et al., 2006; Deshpande and Patla, 2007; Maitre et al., 2013a,b, 2015; Eikema et al., 2014; Maitre and Paillard, 2016). The main objective of these techniques was to alter or manipulate sensory information (i.e., afferents emanating from the visual, vestibular and somatosensory systems) in order to analyze postural compensatory strategies, and to understand how individuals cope so that they can reorganize their posture in a challenging sensory context. Although cutaneous, proprioceptive and visual sensory manipulations have been frequently used to study the relationship between aging effects and physical activity effects on the postural system (Gauchard et al., 2003; Hue et al., 2004; Maitre et al., 2013b), this relationship has rarely been studied in a context where the vestibular afferent signal is manipulated (Maitre et al., 2015).

The integration of vestibular afferents enables the control of eye movements, stabilization of the gaze, and the perception of head orientation, contributing to the control of posture and balance (Cullen, 2012). The artificial alteration of the vestibular afferent signal induces an erroneous perception of head movement (i.e., illusion of motion), which generates an inadequate motor response (Fitzpatrick and Day, 2004; St George and Fitzpatrick, 2011). To analyze postural compensatory strategies in a context of a vestibular sensory manipulation, galvanic vestibular stimulation (GVS) has been widely used (Wardman and Fitzpatrick, 2002; Wardman et al., 2003; Balter et al., 2004a; Fitzpatrick and Day, 2004; Cenciarini and Peterka, 2006; Deshpande and Patla, 2007; Fransson et al., 2007; St George and Fitzpatrick, 2011; Maitre et al., 2013a, 2015; Eikema et al., 2014; Héroux et al., 2015; Yang et al., 2015; Maitre and Paillard, 2016). The GVS delivered a transmastoid electrical current to modulate the continuous firing level of vestibular afferents in order to generate a fictitious vestibular signal of head movement (Wardman and Fitzpatrick, 2002; St George and Fitzpatrick, 2011). The GVS technique generates a stereotyped postural response from the whole body depending on the GVS design, the current intensity, the postural task and the availability of other sensory information (Fitzpatrick and Day, 2004).

Among these previous studies about GVS, few have focused on how older individuals reorganize their posture (Balter et al., 2004b; Deshpande and Patla, 2007; Maitre et al., 2013a, 2015; Eikema et al., 2014) and how the effects of regular physical activity alter the individuals’ postural response to GVS (Balter et al., 2004b; Yang et al., 2015; Maitre and Paillard, 2016). In addition, only one study discussed the way older individuals adapt their postural control to the effects of GVS, in relation to their physical activity status (Maitre et al., 2015). Previous studies indicated that the magnitude of the postural responses to a fictitious vestibular signal (i.e., induced by means of the GVS technique) differed between physically active individuals and non-active individuals (Balter et al., 2004b; Yang et al., 2015; Maitre and Paillard, 2016). Nevertheless, the participants in these studies had not suffered from the involution induced by aging effects, since they were young. To highlight postural control difference, previous studies suggested that the choice of GVS intensity is important (Maitre et al., 2015; Maitre and Paillard, 2016). Maitre et al. (2015) reported no differences between active and non-active older adult groups with a current intensity of 1 mA. However, in another study, they reported differences between active and non-active young adult groups with a current intensity of 3 mA (Maitre and Paillard, 2016).

On the basis of the data mentioned above, to highlight the effects of the regular practice of physical activity on older individuals’ postural system, it would be interesting to focus on how older individuals withstand a 3 mA vestibular perturbation. We hypothesized that the magnitude of the postural control impairment due to GVS would be attenuated by the regular practice of physical activity in older individuals compared to non-active older individuals.

## Materials and Methods

### Participants

After interviewing each volunteer and a medical examination, two groups of healthy older women (*n* = 34) participated in the study. This experimental procedure received the approval of the local committee for the protection of human subjects (Comité de Protection des Personnes Sud-Ouest et Outre Mer I; approval number ID RCB: 2009-A00135-52) and all participants gave written informed consent. The participants were free from any disorders (i.e., neurological, motor and metabolic disorders) or medical conditions that might affect postural control. More precisely, participants were free from any vestibular disorders and did not present any dizziness or vertigo. All the participants led independent lives. This cohort comprised one group of 17 physically active participants (the active group) and one group of 17 non-active participants (the non-active group). To be included in the active group, participants had to have regularly performed gentle physical activity (equal to or more than 3 h a week) in a sports club (i.e., soft gym, aquarobic, active walking, ballroom dancing) for at least 3 years. To be included in the non-active group, participants had to have not practiced physical activity (at home or in a sports club) for at least 3 years except for daily tasks.

### Measurements

To analyze the postural challenge imposed by the GVS, the postural control of each participant was assessed once in three postural conditions, lasting 20 s. Each participant was instructed to stand up in a barefoot bipodal position (i.e., arms across their trunk, feet at an angle of 30° according to precise marks, and an inter-malleolar distance of 9 cm) on a force platform (Techno Concept™, Mane, France; 40 Hz frequency, 12 bit A/D conversion). They were instructed to keep their eyes open and fixed on a target (4 cm^2^) 1.5 m in front of them at the height of their eyes, and to remain as still as possible. Posturowin software (Techno Concept™, Cereste, France) calculated the center of foot pressure (COP) displacement parameters: the COP surface (mm^2^), the COP velocity (mm.s^−1^) detailed on the anteroposterior (COP_Y_ velocity) and the mediolateral (COP_X_ velocity) directions, the maximal amplitude (mm) detailed on the anteroposterior (COP amplitude Y) and the mediolateral (COP amplitude X) directions (Paillard and Noé, 2015).

To avoid the learning effect of the postural task (i.e., quiet stance with eyes open, as still as possible on the force platform) between the different conditions, the participants underwent one trial on the force platform in real evaluation conditions prior to recording. Participants underwent once three postural tests. They were tested in a reference condition (REF condition, i.e., quiet stance) and two randomized GVS conditions. The GVS technique can be considered to modulate the hyperpolarization of the neuroepithelia of the cristae and maculae (Fitzpatrick and Day, 2004). Cathodal currents depolarize and thus increase the firing rate of the vestibular afferents whereas anodal currents hyperpolarize and decrease the firing rate (Wardman and Fitzpatrick, 2002). The GVS technique was used to alter the vestibular information inducing a fictitious asymmetry of vestibular afferences throughout the entire duration of the data recording (Fitzpatrick and Day, 2004; St George and Fitzpatrick, 2011) by means of a 3 mA transmastoid current delivered by a constant-current stimulator (Galvadyn 2, Electronic Conseil, Gallargues le Montueux, France) through electrodes placed over the mastoid bones. An anesthetic gel was applied to each mastoid process to avoid any local noxious sensation. The GVS was set up in a range of 5 s before the recording of postural sway data to avoid recording initial transients and anticipation behavior at the onset. The GVS designs corresponded to two binaural bipolar designs which generate a body tilt in the direction of the anode: the GVS-R condition (i.e., anode placed on the right mastoid process and cathode placed on the left mastoid process) and the GVS-L condition (i.e., anode placed on the left mastoid process and cathode placed on the right mastoid process).

The absolute increases between the REF condition and the GVS conditions were calculated for all the COP parameters.

Absolute increase = GVS condition − REF condition

### Statistical Analysis

Statistical analyses were performed with R statistical software. The normal distribution of data was checked using the Shapiro-Wilk test. Because normality was skewed in most cases, non-parametric statistical tests were used. The age and the anthropometrical data were compared using the Mann-Whitney tests for unpaired data. Paired-samples Wilcoxon signed-rank tests were performed to determine whether there were differences between REF and the GVS-L or GVS-R conditions for all the temporal and/or spatial parameters of the COP displacements. To determine whether there were differences between the active and non-active groups and whether there were differences between the GVS-R and the GVS-L conditions in terms of all the temporal and/or spatial parameters of the COP displacements and the absolute increase, the Mann-Whitney tests for unpaired data were performed.

Results were considered significant at the level of 5%.

## Results

### Age and Anthropometrical Data

The medians and interquartile ranges (IQR) for age, height, weight and foot size for the active and the non-active groups are presented in Table 1. There is no difference between the active and non-active groups (Table 1).

**Table 1:** Medians and interquartile ranges (IQR) of the age and the anthropometrical data for the active and non-active groups.

	Active	Non-active
Age (years)	73.4 (5.8)	73.7 (8.1)
Height (cm)	155.0 (5.0)	155.0 (9.0)
Weight (kg)	65.0 (9.0)	62.0 (11.0)
Foot size (cm)	26.0 (1.3)	25.3 (1.3)

### Postural Control Parameters

**Figure 1:**
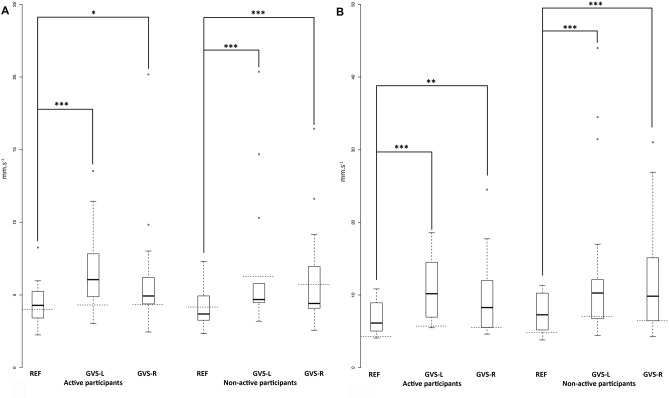
The center of foot pressure (COP)_X_ velocity **(A)** and the COP_Y_ velocity **(B)** in the reference and the galvanic vestibular stimulation (GVS) conditions for the active and non-active groups. *Indicates *p* < 0.05. **Indicates *p* < 0.01. ***Indicates *p* < 0.001. The horizontal broken lines indicate median values for young participants from Maitre and Paillard (2016).

**Figure 2:**
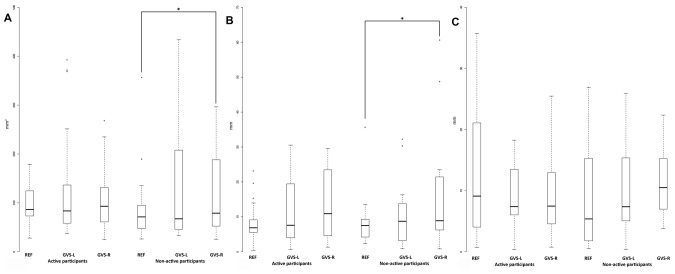
The COP surface **(A)**, the COP_X_ amplitude **(B)** and the COP_Y_ amplitude **(C)** in the reference and the GVS conditions for the active and non-active groups. ^*^Indicates *p* < 0.05.

The medians and IQR for the postural control parameters (i.e., COP surface, COP_X_ and COP_Y_ velocities, COP_X_ and COP_Y_ amplitudes) are presented in Figures 1, 2. The medians and IQR for the absolute increases of the postural control parameters are presented in Table 2. The horizontal broken lines on the Figure 1 and the values between square brackets in the Table 2 indicate median values for young participants corresponding to the same physical activity status in the same conditions, extracted from Maitre and Paillard (2016).

**Table 2:** Absolute increase medians (IQR) of the center of foot pressure displacements parameters of the galvanic vestibular stimulation (GVS) conditions from the reference condition for the active and non-active groups.

	Active	Non-active	Mann-Whitney tests
**COP Surface**			
GVS—R	6.6 (73.3)	9.3 (54.3)	NS
GVS—L	16.3 (122.5)	5.5 (60.6)	NS
Wilcoxon tests	NS	NS
**COP_X_ velocity**			
GVS—R	1.2 (1.9) [0.7]	1.4 (1.5) [2.5]	NS
GVS—L	2.3 (1.9) [0.5]	1.3 (1.5) [1.4]	NS
Wilcoxon tests	NS	NS
**COP_Y_ velocity**
GVS—R	1.2 (5.1) [0.8]	1.7 (5.4) [2.0]	NS
GVS—L	3.4 (3.6) [0.9]	2.5 (4.3) [1.6]	NS
Wilcoxon tests	NS	NS
**COP_X_ amplitude**
GVS—R	2.7 (12.5)	4.1 (10.2)	NS
GVS—L	2.0 (12.1)	−0.6 (5.4)	NS
Wilcoxon tests	NS	NS
**COP_Y_ amplitude**
GVS—R	0.2 (19.9)	2.9 (14.0)	NS
GVS—L	−4.1 (13.3)	1.3 (8.3)	NS
Wilcoxon tests	NS	NS

*Note: NS indicates non-significant difference. [ ] Indicates median absolute increase values for young participants from Maitre and Paillard (2016)*.

There was no significant difference between the two groups in all the conditions (Table 2; Figures 1, 2). The COP_X_ and COP_Y_ velocities significantly increased in the GVS-R and the GVS-L conditions compared to the REF condition for the active and non-active groups (Figure 1). The COP surface and the COP_X_ amplitude increased significantly in the GVS-R condition compared to the REF condition for the non-active group (Figure 2).

## Discussion

The aim of this study was to compare the ability of older individuals to maintain an efficient upright stance in contexts of vestibular sensory manipulations (i.e., GVS), depending on their physical activity status (i.e., active and non-active). The results did not support our hypothesis since there was no difference between the active and non-actives groups in all the conditions. More precisely, although the GVS altered postural control for both groups, the active group did not demonstrate more efficient postural control than the non-active group in a context where the vestibular system was artificially stimulated.

In the present study, the COP_X_ and COP_Y_ velocities increased significantly between the REF condition and the GVS conditions for both groups. Indeed, GVS modulates the continuous firing level of vestibular afferents, producing a potent effect on body motor control as a whole (Fitzpatrick and Day, 2004). This sensory manipulation (i.e., bipolar binaural GVS) induces an artificial signal of head movement which produces a stereotyped postural response, mainly in the mediolateral direction. This erroneous vestibular signal conflicts with the afferents signal emanating from other sensory systems, forcing CNS to generate a counteracting postural response in order to preserve balance.

Furthermore, the COP surface and the COP_X_ amplitude significantly increased between the REF condition and the GVS-R condition for the non-active group. The significant results obtained for the GVS-R condition and not for the GVS-L condition might suggest that there were different GVS effects depending on the GVS design. However, there was no difference between the GVS-R and the GVS-L conditions including for absolute increases. Hence, there was no vestibular prevalence identified in this study. Otherwise, the most intriguing result in this study is that there was no difference between the two groups according to their physical activity status. The significant difference observed between the REF condition and the GVS-R condition for the non-active group but not for the active group might suggest that there were different GVS effects depending on the physical activity status. However, there was no group difference in any condition. In addition, there was no difference concerning the absolute increase. Hence, the active group did not demonstrate a more efficient postural control than the non-active group in a context where vestibular afferents were manipulated. Several responses might explain this result.

First, previous studies (Balter et al., 2004b; Yang et al., 2015; Maitre and Paillard, 2016) indicated that chronic repeated acceleration and deceleration of the head may generate adaptation and habituation mechanisms which reduce the influence of GVS on postural control. The physiological acceleration and deceleration of the head may provoke an erroneous perception of the movement of the head, linked to the inertia of the endolymph and the macula. Repetitive exposure to this stimulus induces a habituation mechanism, which enhances the ability to modulate the amplitude and duration of the disturbing effects of erroneous vestibular signals (Grunfeld et al., 2000; Quarck and Denise, 2005; Deveze et al., 2014). These adaptation and habituation mechanisms resulted from important/intense vestibular stimulations (i.e., translational acceleration and/or angular acceleration of the head) through sports activity (e.g., tumble turn in natation, somersault in gymnastic, brutal head rotation in collective sports) or plane flight activity. Hence, individuals may develop the ability to reduce the postural effects of discordant vestibular afferents in relation to other sensory afferents. In the present study, the active group appeared unable to take advantage of their experience of vestibular stimulation to alleviate the effects of GVS on postural control more efficiently than the non-active group. Conversely to previous studies (Balter et al., 2004b; Yang et al., 2015; Maitre and Paillard, 2016), in this study, the participants practiced gentle physical activities (i.e., soft gym, aquarobic, active walking, ballroom dancing) which solicit the vestibular system less intensely. Soft gym, aquarobic, active walking and ballroom dancing are physical activities suited to a specific public (i.e., older individuals). They do not involve systematic rapid head movements and so do not stimulate the vestibular system strongly. It can be suggested that, although the participants in this study practiced physical activity on a regular basis, the vestibular stimulation resulting from their gentle physical practice was not strong enough to develop their ability to withstand a vestibular sensory manipulation compared to the younger participants in previous studies (Balter et al., 2004b; Yang et al., 2015; Maitre and Paillard, 2016). This could explain why there was no difference between the two groups in terms of GVS conditions.

Second, GVS induces an erroneous vestibular signal, which conflicts with afferent signals emanating from other sensory systems. In a context where one or more sensory systems give an erroneous signal, the CNS triggers a mechanism to adjust the sensory contributions of each sensory system in order to preserve postural control (Massion, 1994; Jeka et al., 2006). Regular physical activity may develop the specific ability to reweight sensory channels appropriately or to switch from one sensory channel to another one that is better adapted to the postural condition induced by the sensory manipulation (Vuillerme et al., 2001; Maitre et al., 2013b; Hopper et al., 2014; Paillard, 2017). In the present study, the active group appeared unable to take advantage of other available sensory information to counteract the disruptive effect of GVS on postural control more efficiently than the non-active group. Furthermore, aging involutions of the structure involved in the postural control induced appear unavoidable (Sturnieks et al., 2008) and the aging process may interfere with the ability of the individual to adapt to physical training, which could contribute to lessening the adaptation induced by regular physical activity (Paillard, 2009). Although physical activity may enhance the use of sensory afferents (Gauchard et al., 2001, 2003; Ribeiro and Oliveira, 2007), owing to the age of the participants, the weak head acceleration and the relatively low intensity induced by the physical activity adapted to older individuals, the active group might have not developed their ability to withstand an unexpected vestibular manipulation more efficiently than the non-active group.

Third, another explanation, which may complete the previous ones, could be linked to the central processing of the vestibular afferents. Indeed, the vestibular system undergoes structural and functional involution with increasing age. Aging is associated with a progressive alteration of the vestibular neuroepithelium (e.g., decrease in the number of hair cells and primary afferent fibers) which may alter vestibular sensitivity (Matheson et al., 1999; Rauch et al., 2001). Nevertheless, previous studies have highlighted central compensatory neural mechanisms to counteract the anatomical loss of hair cell receptors and primary afferents in order to preserve vestibular function (Jahn et al., 2003; Peters et al., 2016). These adaptive neural mechanisms render older individuals more sensitive to electrical vestibular stimuli than younger individuals (Peters et al., 2016). Previous studies (Deshpande and Patla, 2007; Maitre et al., 2013a) have indicated greater effects of GVS on the postural control of older individuals than on that of younger individuals. Unlike other studies involving young participants (Balter et al., 2004b; Yang et al., 2015; Maitre and Paillard, 2016), the greater effect of GVS on older individuals could have reduced the difference between the active and non-active older groups in the present study. Otherwise, Welgampola and Colebatch (2001) and Jahn et al. (2003) reported that beyond the sixth decade the GVS effect may be not constant in magnitude for older individuals. This may increase the interindividual variability response to the GVS in older participants, which constitutes a confounding factor in this study that also may reduce the difference between the two groups.

This study presents several limitations. First, the postural task might not be challenging enough to reveal any difference according to physical activity status. Bipedal postural tasks with the eyes open, on a stable surface and in a stable environment might be too simple to produce evidence of differences between active and non-active participants. Indeed, the more difficult the postural task is and the more specific it is to the physical practice, the greater the difference in postural control between active and non-active individuals (Paillard, 2017). Second, the ability to maintain balance may be specific to the physical activity practiced (Paillard, 2017). In the present study, the participants included in the active group do not practice the same physical activity. Since the specific practice of a physical activity may develop dependence on a sensory system (Paillard, 2017), the effect of GVS might differ depending on the type of physical activity. Third, the sample size was relatively small which may be possibly insufficient to achieve significant differences. Fourth, beyond the sixth decade the GVS may be not the more efficient manipulation technique to reveal postural difference between the two older groups of different physical status.

## Conclusion

In the present study, the main result indicated that there was no difference between the active group and the non-active group. It is likely that the aging process and the type of physical practice had limited the ability of the active group to counteract GVS effects on postural control more efficiently than the non-active group. The active group appeared unable to take advantage of their experience of vestibular stimulation and/or of other available sensory information to alleviate the GVS effects on postural control more efficiently than the non-active group. In the present study, the characteristics of the physical practice appear not to be challenging enough for the vestibular system to develop the active group’s ability to withstand an unexpected vestibular signal more efficiently than the non-active group. In addition, the aging process may have altered the effect of the physical activity program and the manner of integrating the vestibular signal, thereby contributing to reducing the difference between the two groups.

## Author Contributions

JM and TP contributed to the conception and design of the work; and the acquisition, analysis and interpretation of data.

## Conflict of Interest Statement

The authors declare that the research was conducted in the absence of any commercial or financial relationships that could be construed as a potential conflict of interest.
